# Predicting Intrinsic Motivation After an Adventure Education Program in Primary Schools: Enjoyment, Self-Confidence and Resilience According to Gender

**DOI:** 10.3390/bs16060874

**Published:** 2026-06-01

**Authors:** Andrés Calmaestra-Sánchez, Antonio Baena-Extremera, Josué González-Ruiz, José Antonio Sánchez-Fuentes

**Affiliations:** 1 Department of Didactics of Musical, Plastic and Bodily Expression, University of Granada, 18015 Granada, Spain; andrescalmaestra@gmail.com (A.C.-S.); abaenaextrem@ugr.es (A.B.-E.); 2 Research Group Movement Sciences and Sport (MS&SPORT), Department of Physical Activity and Sport, Faculty of Sport Sciences, University of Murcia, 30720 Murcia, Spain

**Keywords:** adventure education, motivation, parkour

## Abstract

This study aimed to describe the pre–post changes in intrinsic motivation observed following the implementation of a parkour-based Adventure Education (AE) program in primary school students, and to examine the role of enjoyment, self-confidence, and resilience as variables statistically associated with intrinsic motivation, considering differences according to time (pre-test–post-test) and gender. The sample consisted of 492 fifth- and sixth-grade primary education students (249 boys and 243 girls) with a mean age of 10.67 years, enrolled in 12 Spanish schools. A quasi-experimental design with pre-test and post-test measures was used following the implementation of a seven-session program based on the Pedagogical Model of Adventure Education. Data were collected using instruments validated in the Spanish population: the intrinsic motivation subscale of the Perceived Locus of Causality Scale, the Physical Activity Enjoyment Scale to measure enjoyment, the self-confidence subscale of the Competitive State Anxiety Inventory, and the Connor–Davidson Resilience Scale to assess resilience. Statistical analysis was performed using SPSS 28.0 software, conducting descriptive analyses, correlations, and hierarchical multiple linear regressions to examine the statistical associations among the variables at each measurement point, along with a 2 × 2 repeated-measures ANOVA (time × gender). Post-test scores were significantly higher than pre-test scores for intrinsic motivation, enjoyment, self-confidence and resilience. Enjoyment was the variable most strongly statistically associated with intrinsic motivation, followed by self-confidence and resilience. The ANOVA showed a significant main effect of time, while no significant time × gender interaction was detected, meaning that the study found no evidence that the pre–post change differed between boys and girls. Given the single-arm pre–post design and the absence of a control group, these findings should be interpreted as preliminary descriptive evidence of pre–post change and associations, and not as a causal test of the program’s effectiveness.

## 1. Introduction

In the field of Physical Education (PE), motivation is recognized as a key factor for ensuring students’ enjoyment, active participation, and the promotion of long-term healthy habits, habits that international agencies such as the World Health Organization (2024) consider essential for child and adolescent health (Becerra-Fernández et al., 2025). The contributions of Self-Determination Theory (SDT; Deci & Ryan, 2000) distinguish, among other things, between intrinsic motivation (engaging in an activity for inherent pleasure and satisfaction) and extrinsic motivation (participating because of external rewards or internal pressures). This distinction has recently been applied within PE and has shown that intrinsic motivation is associated with positive achievement-related emotions, such as greater enjoyment and persistence (Carson et al., 2025; Navarro-Patón et al., 2024) in both boys and girls, whereas amotivation is linked to negative emotions and withdrawal from physical activity (Işıkgöz, 2025) in both genders. Furthermore, regarding the students’ gender, some studies have reported higher levels of intrinsic motivation and enjoyment among boys compared to girls (Navarro-Patón et al., 2024; Gómez-Rijo et al., 2011; Botella et al., 2021).

In recent years, methodologies have emerged that enhance motivation while seeking to foster more meaningful learning and more positive emotions in Physical Education (PE). The Pedagogical Model of Adventure Education (PMAE) is characterized by individual, cooperative, and experiential tasks that make students face physical, social, and emotional challenges—combining challenge, uncertainty, adventure, and learning within the PE lesson (González-Melero et al., 2026). A recent mixed review shows that Adventure Education (AE) programs improve resilience and life satisfaction while reducing stress levels; moreover, they promote leadership, self-efficacy, peer support, and a sense of group belonging (Ghani et al., 2025). Using this pedagogical model, successful practices have been reported over a wide range of psychological and academic variables, based on activities such as caving, climbing, via ferratas, and numerous adventure sports (Baena-Extremera et al., 2012; González-Melero et al., 2026; Horno-Tomé et al., 2025), with parkour emerging as one of these practices.

The practice of parkour, although popularized in France during the 1990s by David Belle and a small group of practitioners, has its conceptual roots in earlier movement traditions. In particular, the discipline draws on the méthode naturelle developed by the French physical educator Georges Hébert in the early twentieth century, who advocated for training the body through running, jumping, climbing, balancing, and lifting in natural environments. This same locomotor repertoire was later codified in obstacle and challenge course traditions that have informed Adventure Education for decades (Atkinson, 2009). From this lineage, parkour has evolved into a non-competitive practice that combines running, jumping, and climbing skills, and that fits well within the Adventure Education framework. As with other AE contents, parkour places students in unfamiliar physical situations, involves a degree of perceived risk, and requires problem-solving and the cooperative negotiation of obstacles (Baena-Extremera et al., 2012; Ghani et al., 2025; González-Melero et al., 2026). Its creative nature and emphasis on self-determination make it a discipline that can facilitate the satisfaction of autonomy and competence needs (Miranda-Ullán et al., 2024). A recent multimethod analysis highlighted how the non-competitive and creative nature of parkour can support intrinsic motivation in young people, offering an environment in which exploration, self-expression, and camaraderie foster sustained participation (Carson et al., 2025). However, the literature on parkour within school contexts is still limited (Montoro & Baena-Extremera, 2015); most studies focus on extracurricular programs and adolescents, and there is a clear lack of empirical research on primary school students (Torronteras, 2023), whether in Spain or internationally. Even fewer studies exist that combine parkour with such an experiential and innovative pedagogical model.

Given the current situation, the following research hypotheses have been formulated for this work:

**Hypothesis 1** **(H1).** *After participating in the parkour-based AE program, students will show significantly higher post-test scores than pre-test scores in intrinsic motivation, enjoyment, self-confidence and resilience*.

**Hypothesis 2** **(H2).** *Intrinsic motivation will be positively and significantly associated with enjoyment, self-confidence, and resilience, both before and after the intervention, in boys and girls*.

**Hypothesis 3** **(H3).** *Enjoyment will be the variable most strongly statistically associated with intrinsic motivation, followed by self-confidence and resilience, both at pre-test and post-test, regardless of gender*.

**Hypothesis 4** **(H4).** *Mean intrinsic motivation scores will be significantly higher at post-test than at pre-test*.

Considering these hypotheses, the aim of this research was to describe the pre–post changes observed in intrinsic motivation following the implementation of a parkour-based Adventure Education (AE) program in primary school students, and to examine the role of enjoyment, self-confidence and resilience as variables statistically associated with intrinsic motivation, taking into account differences based on time (pre-test–post-test) and gender.

The following specific objectives were established:

1. To describe the pre–post changes observed in the students’ intrinsic motivation, enjoyment, self-confidence and resilience following the implementation of the parkour-based Adventure Education program.
2. To analyze the relationships between intrinsic motivation and enjoyment, self-confidence, and resilience, differentiating by gender and assessment time (pre-test and post-test).
3. To examine the statistical associations of enjoyment, self-confidence and resilience with intrinsic motivation at pre-test and at post-test, differentiating between boys and girls.
4. To test, through a repeated-measures analysis, whether mean intrinsic motivation scores differed between pre-test and post-test and whether that pre–post difference varied by gender.

## 2. Materials and Methods

### 2.1. Sample

The sample design was non-probabilistic and, by convenience, based on subjects who were accessible. The sample consisted of 492 students (249 boys, 50.6%; 243 girls, 49.4%) with a mean age (*M*) of 10.67 years and a standard deviation (*SD*) of 0.546. Of these, 180 were 10 years old (36.6%), 293 were 11 years old (59.6%), and 19 students were 12 years old (3.9%), with 147 students (29.9%) from the 5th grade and 345 (70.1%) from the 6th grade of primary education. The students came from 12 Spanish schools (in Córdoba, Cádiz, Zaragoza, Segovia, Alcorcón, Navarra, Toledo, and Palencia) (see Table 1).

Regarding the sample size of the present study (*N* = 492), this can be considered sufficient and adequate for performing the statistical analyses. According to the methodological recommendations for correlational analyses and multiple linear regression with moderate effect sizes (*f^2^* ≈ 0.15), a significance level of *α* = 0.05 and a minimum statistical power of 0.80 is desirable—the required sample size is therefore considerably lower than that used in this study. Similarly, for a repeated-measures ANOVA with one within-subjects factor (time) and one between-subjects factor (gender), assuming a medium effect size (*η^2^p* ≈ 0.06), the recommended sample size would be less than 200 participants (Miranda, 2025; Ryan, 2013). In this regard, the sample employed in the present study can be considered adequate for the planned analyses. It should nevertheless be noted that the data have a clustered structure (students nested within classes and schools) that was not modeled analytically; precision estimates may therefore be affected by this unmodelled clustering, an issue that is addressed in the Section 5.

### 2.2. Design

A single-arm pre–post quasi-experimental design was used for the intervention component of the study, with intrinsic motivation, enjoyment, self-confidence and resilience assessed before and after the program. In addition, descriptive and correlational analyses, as well as hierarchical multiple linear regressions, were carried out cross-sectionally within each measurement point (pre-test and post-test) in order to describe the statistical associations among the variables; these analyses are therefore associative rather than predictive in a temporal or causal sense. No control group was included, and assignment of participants was non-random; both aspects are explicitly considered in the Section 5. The organization of the schools and classrooms was respected throughout the study. To conduct this study, several school and student selection criteria were set: (1) schools willing to implement an Adventure Education (AE) program in the classroom; (2) schools willing to allow students to leave the school premises during the first term as many times as necessary to complete the proposed program; (3) schools with the capacity to include a parkour-related learning situation in their teaching plans using the Pedagogical Model of Adventure Education (PMAE). Based on these criteria, 12 schools from different geographical areas of Spain were selected (see Table 1).

### 2.3. Instruments

Various instruments that had been previously validated in the Spanish population were employed for data collection and to evaluate the variables under study:

Intrinsic Motivation: The intrinsic motivation variable from the Perceived Locus of Causality Scale in Physical Education (PLOC Scale) by Goudas et al. (1994), adapted by Moreno et al. (2009), was used. The instrument is preceded by the phrase “I participate in this Physical Education class …” and uses items such as “…because Physical Education is fun,” in which students respond on a Likert scale from 1 (totally disagree) to 7 (totally agree). The fit and reliability indices in the pre-test and post-test were as follows: Cronbach’s alpha (α) = 0.70/0.70, McDonald’s omega composite reliability (ω) = 0.72/0.73, and average variance extracted (AVE) = 0.52/0.57.

Enjoyment: The enjoyment variable from the Physical Activity Enjoyment Scale (PACES) by Motl et al. (2001), adapted by Moreno et al. (2008), was used. This instrument begins with the phrase “When I am active …” and uses items of the type “… I enjoy it,” where students must respond on a Likert-type scale from 1 (totally disagree) to 5 (totally agree). The fit and reliability indices in the pre-test and post-test were α = 0.78/0.81, ω = 0.74/0.78, and AVE = 0.61/0.66.

Self-Confidence: To measure self-confidence, the self-confidence variable from the Spanish version of the Competitive State Anxiety Inventory CSAI-2R by Andrade et al. (2007) was used. This inventory integrates a self-confidence subscale that estimates the degree of security that the subject believes they have regarding their possibilities of success in performing tasks, with items such as “I am confident in myself.” For this purpose, five items are used, which provide a global score between 5 and 20, responding to each of the statements using a Likert-type response format with four alternatives, where 1 is totally disagree and 4 is totally agree. The fit and reliability values in the pre-test and post-test were α = 0.80/0.77, ω = 0.83/0.81, and AVE = 0.65/0.64.

Resilience: Finally, to measure resilience, the CD-RISC scale adapted by Notario-Pacheco et al. (2011) in its Spanish version was used. This measures an individual’s capacity for adaptation and recovery in adverse situations (“In difficult times, I generally expect the best”). The items comprising this instrument are responded to using a Likert scale with five response options, where 0 is never and 4 is almost always. This instrument obtained α = 0.73/0.78, ω = 0.99/0.99, and AVE = 0.97/0.97 in the pre-test and post-test, respectively.

### 2.4. Procedure

Permission was obtained from the competent authorities to conduct the research, both from the primary schools and from the university institutions. The research was carried out in accordance with the 1961 Declaration of Helsinki, with approval from the University of Granada Ethics Committee (UGR), identification number 5306/CEIH/2025.

In this regard, all study participants, or their legal guardians, signed an informed consent form; this provided them with detailed information about the objectives, procedures, and characteristics of the research. They were also informed that their participation was voluntary and that they could withdraw from the study at any time without having any repercussions on their academic grades or educational progress. They were informed that there were no correct or incorrect answers and were asked at the outset to respond with maximum sincerity and honesty. The instruments measuring the different variables were administered in the classroom by the teachers themselves, following a common written protocol provided by the research team. The research team did not deliver the questionnaires directly but coordinated and supervised the administration process across the 12 schools in order to ensure procedural consistency. The potential influence of teacher presence on the students’ responses is acknowledged in the Section 5.

### 2.5. Intervention Program

Prior to the program starting, the pre-intervention questionnaires were administered by each teacher in their respective school on a mass scale. Once data collection was completed, it was verified that the questionnaires had been correctly filled in. Subsequently, after the program activity had ended, the questionnaires were administered again (the post-intervention), following the same initial procedure, also under the teachers’ supervision to ensure data validity and avoid possible errors.

The program was carried out during the second term of 2025, following the Pedagogical Model of Adventure Education (PMAE) by Baena-Extremera (2011). The description of the intervention can be found below, according to the TIDieR guide (Hoffmann et al., 2014). The objective of the program was to describe the pre–post changes observed in intrinsic motivation, enjoyment, self-confidence and resilience following the implementation of a parkour-based Adventure Education (AE) program. This program is designed for a learning situation consisting of a total of seven sessions. The intervention was applied to each of the groups in the selected schools, with pre- and post-analyses conducted. The intervention is detailed below:

Description of the Intervention Program

1. Name: Adventure Education Program through Parkour (PEAP).
2. Why: Adventure Education is an effective pedagogical approach for improving several variables affecting primary education students, as supported by research conducted in other population contexts.
3. What (materials): the following assessment instruments were employed:
  - PLOC Scale to measure intrinsic motivation;
  - PACES Scale to measure enjoyment;
  - CSAI-2R Scale to measure self-confidence;
  - CD-RISC10 Scale to measure resilience.
4. What (procedures): An intervention program was implemented with measurements prior to and following its execution.
5. Who (provided): The program activities were conducted by teachers specialized in Physical Education; they themselves carried out the pre- and post-assessments.
6. How: The program was delivered through seven structured sessions of 50–55 min each, integrated into the regular Physical Education timetable. The same seven-session structure was applied to all participating groups in the 12 schools, regardless of grade (5th or 6th), in order to ensure consistency of dose across sites and age groups.
7. Where: Both the program activities and the assessments were carried out in 12 Spanish schools (Córdoba, Cádiz, Zaragoza, Segovia, Alcorcón, Navarra, Toledo, and Palencia).
8. When and how much: The program was delivered during the second academic term of 2025 and consisted of seven sessions per group. Pre-test measures were administered one hour before the first session and post-test measures one hour after the seventh session, following the same procedure in every participating school.
9. Adaptation considerations: Each session included a series of activities adapted to the Adventure Education model and according to its phases and stages.
10. Modifications: Adjustments were made to certain activities based on the participants’ level, with safety always the priority.
11. How well planned: The general coordination was the responsibility of the teaching staff who were specialized in implementing the program, and in collaboration with the researchers responsible for the study.
12. How well was it actually carried out: The program was carried out as planned, and implementation was monitored through session checklists. Specifically, each session was guided by a common written protocol describing the objectives, contents, and tasks to be implemented; the teachers in charge completed a brief checklist after every session, recording whether the planned activities had been carried out, the duration of each phase, and any incident or adaptation introduced. These checklists were periodically reviewed by the research team in order to monitor cross-site consistency. It must nevertheless be acknowledged that delivery was distributed across 12 schools and several teachers, which inevitably introduces some variability in instructional style and minor adjustments, even though the protocol was the same for every group.

### 2.6. Statistical Analysis

Data analysis was conducted using IBM SPSS Statistics (version 28.0) software. First, normality tests and descriptive analyses of all the variables under study were performed, calculating the means (M), standard deviations (SD), and the skewness and kurtosis indices to examine the data distribution and to verify their suitability for parametric or non-parametric analyses.

The internal consistency of each instrument was analyzed using various coefficients, including Cronbach’s alpha, McDonald’s omega, and the average variance extracted (AVE). The reliability criteria were values above 0.70, and AVE was greater than 0.50 (Hair et al., 2019).

After confirming the data as being “non-normal,” Spearman’s bivariate correlation analyses were conducted to examine the relationships between intrinsic motivation, enjoyment, self-confidence, and resilience, differentiated by gender (boys and girls) and assessment time (pre-test and post-test). Subsequently, a hierarchical multiple linear regression analysis was performed cross-sectionally at each measurement point to describe the statistical associations of enjoyment, self-confidence and resilience with intrinsic motivation, which was treated as the criterion variable. Because predictors and the criterion variable were assessed simultaneously within each wave, these regression analyses are associative rather than predictive in a temporal or causal sense, and the term “predictor” is used hereafter only in the technical sense of “statistical predictor” within the regression model. The analyses were conducted separately for boys and girls, both at pre-test and post-test. Enjoyment was introduced in the first block, self-confidence in the second block, and resilience in the third block. Prior to this, residual independence assumptions were checked using the Durbin–Watson statistic, as well as the absence of multicollinearity using tolerance indices and the variance inflation factor (VIF), with the obtained values being deemed adequate.

Although the normality tests indicated deviations from normality, a 2 × 2 repeated-measures ANOVA was employed due to the sample size (N = 492) and the robustness of ANOVA to moderate deviations from this assumption, especially when group sizes are large. Likewise, non-parametric alternatives (e.g., Friedman/Wilcoxon) do not allow direct evaluation of the time × gender interaction with the same 2 × 2 model design (Hair et al., 2009; Mertler et al., 2025).

## 3. Results

Table 2 and Table 3 present the descriptive statistics (mean, standard deviation, skewness and kurtosis) of the variables analyzed by gender and assessment time (pre- and post-intervention).

For the boys (Table 2), the results show an increase in the means of all the variables following the intervention. Intrinsic motivation went from a mean of 5.84 (SD = 0.79) in the pre-test to 6.34 (SD = 0.82) in the post-test. Similarly, enjoyment increased from 3.96 (SD = 0.58) to 4.35 (SD = 0.55), self-confidence from 3.19 (SD = 0.61) to 3.55 (SD = 0.47), and resilience from 2.87 (SD = 0.50) to 3.13 (SD = 0.58).

The skewness and kurtosis values in the pre-test are close to zero for all the variables, indicating approximately normal distributions. In the post-test, greater negative skewness and higher kurtosis values are observed in intrinsic motivation and self-confidence, suggesting a greater concentration of high scores following the intervention.

For the girls (Table 3), a similar pattern is observed. Intrinsic motivation increased from 5.80 (SD = 0.81) in the pre-test to 6.21 (SD = 0.97) in the post-test. Enjoyment went from 3.92 (SD = 0.53) to 4.30 (SD = 0.58), self-confidence from 3.12 (SD = 0.55) to 3.47 (SD = 0.52), and resilience from 2.85 (SD = 0.45) to 3.04 (SD = 0.65).

As with the boys, the skewness and kurtosis indices in the pre-test reflect distributions close to normality, while, in the post-test, more pronounced negative skewness and positive kurtosis values are observed in intrinsic motivation and self-confidence, indicating a shift in scores towards higher values following the intervention.

### 3.1. Correlation Analysis

Table 4 and Table 5 show the Spearman correlation coefficients between the analyzed variables, differentiated by gender and assessment time. In both tables, the upper part corresponds to the pre-test and the lower part to the post-test.

For the boys (Table 4), intrinsic motivation in the pre-test was positively and significantly related to enjoyment (r = 0.520, *p* < 0.01), self-confidence (r = 0.301, *p* < 0.01), and resilience (r = 0.306, *p* < 0.01). Likewise, significant correlations were observed between enjoyment and self-confidence (r = 0.352, *p* < 0.01), enjoyment and resilience (r = 0.390, *p* < 0.01), and self-confidence and resilience (r = 0.450, *p* < 0.01).

In the post-test, the relationships intensified, especially between intrinsic motivation and enjoyment (r = 0.667, *p* < 0.01). Positive and significant correlations were also observed between intrinsic motivation and self-confidence (r = 0.423, *p* < 0.01) and resilience (r = 0.436, *p* < 0.01). The associations between self-confidence and resilience were high (r = 0.503, *p* < 0.01), indicating a strong interrelationship between these variables following the intervention.

For the girls (Table 5), intrinsic motivation in the pre-test showed positive and significant correlations with enjoyment (r = 0.414, *p* < 0.01), self-confidence (r = 0.220, *p* < 0.01), and resilience (r = 0.218, *p* < 0.01). The relationships between enjoyment and self-confidence (r = 0.160, *p* < 0.01) and between enjoyment and resilience (r = 0.165, *p* < 0.01) were more moderate.

In the post-test, the correlations increased notably. The strong relationship between intrinsic motivation and enjoyment stands out (r = 0.736, *p* < 0.01), as well as with self-confidence (r = 0.538, *p* < 0.01) and resilience (r = 0.542, *p* < 0.01). Additionally, the association between self-confidence and resilience was particularly high (r = 0.661, *p* < 0.01), indicating a strong relationship between both variables following the intervention.

### 3.2. Block Regression Analysis

A hierarchical multiple linear regression analysis was conducted cross-sectionally at each measurement point to describe the statistical associations of enjoyment, self-confidence and resilience with intrinsic motivation, differentiating by gender and assessment time (pre- and post-intervention). The term “predictor” is used here in its technical statistical sense within the regression model and does not imply temporal or causal precedence. Prior to this, compliance with the assumptions of independence and absence of multicollinearity were verified, obtaining adequate tolerance, VIF, and Durbin–Watson statistic values for both the boys and the girls.

The regression analysis results for the boys are presented in Table 6. In the pre-test, enjoyment, which was introduced in the first block, was a significant predictor of intrinsic motivation (ß = 0.710), explaining 27% of the variance (R^2^ = 0.270; F = 91.44, *p* < 0.001). In the second block, self-confidence contributed additionally to the prediction (ß = 0.175), increasing the explained variance to 28%. In the third block, resilience showed a lower weight (ß = 0.132), with the explained variance remaining around 28%.

In the post-test, enjoyment was once again shown to be the variable most strongly statistically associated with intrinsic motivation (ß = 0.997), explaining 44.5% of the variance (R^2^ = 0.445; F = 198.39, *p* < 0.001). The inclusion of self-confidence in the second block increased the explained variance to 46.2%, with a significant weight (ß = 0.283). Finally, the resilience introduced in the third block contributed significantly (ß = 0.173), reaching a total of 47.7% of the explained variance.

The tolerance index for the boys showed pre-test values between 0.736 and 0.876 and post-test values between 0.678 and 0.807. The variance inflation factor (VIF) presented pre-test values between 1.141 and 1.359 and post-test values between 1.239 and 1.474; therefore, these values indicate that the probability of error derived from potential collinearity is ruled out (Hair et al., 2019). Likewise, the obtained Durbin–Watson statistic was between 1.89 in the pre-test and 1.85 in the post-test, confirming the independence of the data (Gil, 2003).

The results for the girls are shown in Table 7. In the pre-test, enjoyment was a significant predictor of intrinsic motivation (ß = 0.635), explaining 17.1% of the variance (R^2^ = 0.171; F = 49.81, *p* < 0.001). In the second block, self-confidence showed a significant weight (ß = 0.233), increasing the explained variance to 20%. In the third block, resilience presented a moderate weight (ß = 0.185), with the explained variance remaining around 20.4%.

In the post-test, enjoyment explained a very high proportion of intrinsic motivation (ß = 0.736), reaching 54% of the explained variance (R^2^ = 0.540; F = 285.14, *p* < 0.001). The incorporation of self-confidence in the second block increased the explained variance to 58.8% (ß = 0.251). Finally, resilience, which was introduced in the third block, showed a significant weight (ß = 0.161), reaching 60% total explained variance.

In the case of the girls, the tolerance index showed pre-test values ranging from 0.774 to 0.975 and post-test values between 0.529 and 0.786. The variance inflation factor (VIF) presented pre-test values between 1.00 and 1.293 and post-test values between 1.273 and 1.889; therefore, these values indicate that the probability of error derived from potential collinearity is ruled out (Hair et al., 2019). Likewise, the Durbin–Watson statistic obtained was between 1.98 in the pre-test and 1.86 in the post-test, confirming the independence of the data (Gil, 2003). Table 6 and Table 7 present the results of the hierarchical linear regression analysis for the boys and the girls in the pre- and post-tests, with intrinsic motivation as the criterion variable.

### 3.3. 2 × 2 Repeated Measures ANOVA

A 2 × 2 repeated-measures ANOVA was carried out, with time (pre-test vs. post-test) as a within-subject factor and gender (boys vs. girls) as a between-subject factor, in order to test whether mean intrinsic motivation scores differed between pre-test and post-test and whether that time difference varied by gender. This analysis tests for differences in means over time and their interaction with gender; it does not test whether the intervention caused those differences, given the absence of a control group(see Table 8 and Table 9).

#### 3.3.1. Model Assumptions

Box’s M test was not significant (M = 7.01, F = 2.33, *p* = 0.072), indicating that the homogeneity of covariance matrices assumption was met. Likewise, Mauchly’s sphericity test was not significant (W = 1.00), so sphericity was assumed for all contrasts. Levene’s test showed homogeneity of variances between genders in the pre-test (*p* = 0.533), although not in the post-test (*p* = 0.027). Nevertheless, given the sample size and the robustness of ANOVA against moderate deviations from this assumption, the analysis proceeded.

#### 3.3.2. Within-Subject Effects

The results showed a significant main effect of time—F(1, 490) = 80.76, *p* < 0.001, and η^2^p = 0.141, with an observed power of 1.00—indicating a significant increase in intrinsic motivation following the intervention. Estimated marginal means reflect an increase from the pre-test (M = 5.82; 95% CI [5.75, 5.89]) to the post-test (M = 6.27; 95% CI [6.19, 6.35]). Pairwise comparisons confirmed that this difference was statistically significant (ΔM = −0.46, *p* < 0.001), and the effect size was large according to standard criteria for partial η^2^.

#### 3.3.3. Time × Gender Interaction

No significant interaction effect was observed between time and gender—F(1, 490) = 0.69, *p* = 0.406, and η^2^p = 0.001—indicating that the increase in intrinsic motivation following the intervention was similar for both the boys and girls. The estimated means show that both groups increased their levels of intrinsic motivation from the pre-test to the post-test (boys: M = 5.84 to M = 6.34; girls: M = 5.79 to M = 6.21).

#### 3.3.4. Inter-Subject Effects

The main effect of gender was not statistically significant—F(1, 490) = 2.37, *p* = 0.124, and η^2^p = 0.005—indicating that no overall differences existed in intrinsic motivation levels between the boys and the girls when both measurement points were considered together. The estimated marginal means were slightly higher for the boys (M = 6.09) than for the girls (M = 6.00), although this difference did not reach statistical significance.

Finally, it should be noted that η^2^p = 0.141 represents a large effect size—this is particularly significant in real-world educational settings. The non-significant time × gender interaction indicates that the study did not detect evidence that the pre–post change differed between boys and girls; this finding should not be interpreted as proof of equivalence or of equal effectiveness across genders, which would require an equivalence or non-inferiority framework with pre-specified margins, ideally within a controlled design.

## 4. Discussion

This research aims to contribute to the scientific knowledge on the current gap in the use and application of innovative methodologies in PE in primary education, providing preliminary descriptive evidence on the pre–post changes and associations observed in connection with the implementation of the Pedagogical Model of Adventure Education (PMAE) with parkour, with respect to intrinsic motivation and its statistically related variables. To this end, it starts from the working assumption that a parkour-based AE program may be associated with higher post-test scores in intrinsic motivation and in related variables such as enjoyment, self-confidence and resilience.

The results obtained in this study suggest that the application of a parkour-based AE program is associated with a significant increase in the students’ intrinsic motivation, enjoyment, self-confidence, and resilience from pre-test to post-test. Given that no control condition was included, however, these changes should not be interpreted as causal effects of the intervention in a strict sense, but rather as changes that coincided with its implementation. The improvement observed in intrinsic motivation suggests that combining the challenging and cooperative activities inherent to AE with the creative approach of parkour offers an environment rich in autonomy and competence—essential elements for fostering self-directed learning. This finding aligns with the postulates of SDT, namely that the satisfaction of basic psychological needs favors self-determined forms of participation and improves persistence in physical activity (Carson et al., 2025; Ghani et al., 2025).

The analysis of predictor variables showed that enjoyment was the variable most strongly associated with intrinsic motivation in both the pre-test and post-test, followed by self-confidence and resilience. A methodological caveat is in order here: the regression analyses were cross-sectional within each measurement point, with predictors and outcome assessed simultaneously, so the present findings describe co-variation among constructs rather than directional or mechanistic relationships. There is also a degree of conceptual proximity between intrinsic motivation and enjoyment within SDT, since inherent enjoyment is itself part of the definition of intrinsic motivation, and the strong association between the two variables therefore partly reflects shared theoretical content rather than evidence that enjoyment causes intrinsic motivation. Bearing all of this in mind, these results align with previous studies highlighting the central role of pleasure and fun in physical participation—when motor experiences are perceived as fun, intrinsic motivation is strengthened and the likelihood of continued practice increases (Işıkgöz, 2025; Ghani et al., 2025). Self-confidence and resilience also contributed significantly; the perception of competence and the ability to overcome challenges are key to persevering when faced with demanding tasks, and, in line with the AE literature, programs that allow for controlled risk-taking increase one’s sense of competence and, consequently, enjoyment (Ghani et al., 2025).

Regarding gender differences, the study found no significant effects on intrinsic motivation or its predictors. This result contrasts with research reporting higher levels of motivation and enjoyment in boys (Navarro-Patón et al., 2024). For instance, in the research by Botella et al. (2021) using parkour and the “flipped learning” methodology, they also showed a greater increase in intrinsic motivation for boys than for girls. The absence of differences could be explained by the cooperative and non-competitive nature of the program, which prioritizes collaboration and personal improvement over competition. AE and parkour offer adaptable activities with low perceived risk that allow girls and boys to participate on equal terms, reducing gender stereotypes and psychological barriers. This finding highlights the potential of these methodologies in promoting inclusive and equitable environments in PE.

## 5. Limitations and Future Prospects

Despite the robustness of the results obtained, one needs to point out certain limitations that were inherent to the study design, and which should therefore be considered when interpreting the findings. Firstly, a quasi-experimental design was used with non-random assignment of participants, in addition to the absence of a control group—this limits the possibility of establishing firm causal relationships between the intervention and the observed changes in the analyzed variables. Without a comparison condition, the pre–post changes cannot be unequivocally attributed to the parkour-based AE program. Alternative explanations cannot be ruled out, including the natural maturation of the students throughout the academic term, repeated exposure to the questionnaires, the novelty of an unfamiliar activity, and other contextual factors linked to the school setting or to the teachers themselves. The present results should therefore be read as preliminary evidence compatible with a positive effect of the program, rather than as a definitive test of its efficacy, and they should serve as a basis for the design of future randomized or controlled studies. Secondly, the program’s duration, which was limited to only seven sessions over one academic term, might be too brief to consolidate profound changes in complex psychological variables such as resilience or self-confidence. The literature indicates that constructs of this nature often require longer intervention periods to generate stable adaptations. Therefore, future studies could incorporate longer programs and longitudinal follow-up measures (for example, post-intervention assessments at several months after the end of the program) to evaluate the stability of the effects in the medium to long term.

A further limitation is that all the study variables were assessed using self-reporting instruments. Although the questionnaires used possess adequate psychometric properties and have been widely utilized in previous research, this type of measurement may be subject to biases associated with social desirability or students’ subjective perceptions, particularly at early educational stages such as in primary education. Consequently, future research could benefit from incorporating mixed-method designs that combine quantitative data with qualitative techniques, such as interviews, systematic observations, or focus groups, to delve deeper into the students’ experience and better understand the underlying processes of motivational improvement.

The implementation context also deserves comment. The program was delivered across 12 schools by different Physical Education teachers, which strengthens ecological validity but also introduces variability in instructional style, possible differences in fidelity between sites, and a clustered structure (students nested within classes and schools) that was not modeled analytically in the present work. Because all 492 students were treated as independent observations and intraclass correlations were not computed in the present work, the standard errors of the analyses may be underestimated and statistical precision may consequently be overstated. The results should therefore be read bearing this caveat in mind, and future replications should explicitly model the clustering by means of multi-level approaches and report intraclass correlations or sensitivity analyses with school or class as a clustering factor. The administration of the questionnaires is also worth commenting on briefly: a common written protocol was followed in all schools, but the instruments were delivered by the teachers themselves in their respective classrooms, and the presence of the teacher may have introduced a potential source of social desirability that cannot be ruled out from the present design. Future studies should consider multi-level approaches, more controlled implementation, and explicit measures of fidelity beyond session checklists. Likewise, parkour requires a minimum of materials and adapted spaces (mats, boxes, benches, parallel bars, urban-style obstacles); not every primary school has direct access to a dedicated parkour course, which may constrain the transferability of the program. The activities used in this study were intentionally designed to be implemented with standard PE equipment in order to ease replication, but the question of access to suitable spaces and materials remains relevant for any large-scale dissemination of this type of intervention.

Finally, as a future line of research, it would be pertinent to analyze in greater depth the relationship of parkour-based AE programs with other relevant indicators of the students’ personal and social development, such as group cohesion, emotional self-regulation, motivational climate, or adherence to physical activity. Similarly, it would be interesting to replicate this type of intervention at different educational stages and in different sociocultural contexts to verify the consistency and generalizability of the pre–post changes and associations observed.

## 6. Conclusions

In relation to the first objective, the results allow one to conclude that the intervention coincided with a significant increase in intrinsic motivation following its application. This finding points to the possibility that the implemented teaching proposal was accompanied by higher self-determined motivation scores toward the practice of PE, although the absence of a control group means that this association cannot yet be interpreted as a causal effect.

Regarding the second objective, post-test scores on intrinsic motivation, enjoyment, self-confidence and resilience were significantly higher than pre-test scores, and scores on these emotional and personal development variables also increased following the program; however, these changes cannot be attributed uniquely to the program, given the absence of a control group and the alternative explanations described in the Section 5. Correlational analyses showed positive and significant associations between intrinsic motivation and enjoyment, self-confidence, and resilience, especially following the intervention. This suggests that the increase in intrinsic motivation is accompanied by a more positive perception of the motor experience and greater personal resources.

With regard to the third objective, regression analyses showed that enjoyment is the variable most strongly associated with intrinsic motivation, followed by self-confidence and resilience, while keeping in mind the conceptual proximity between enjoyment and intrinsic motivation within SDT. This underscores the importance of satisfying and emotionally safe learning contexts. Finally, the non-significant time × gender interaction indicates only that the study did not detect evidence that the pre–post change differed between boys and girls; this finding should not be interpreted as proof that the program is equally effective or equitable across genders.

Overall, the study provides preliminary evidence suggesting that a parkour-based AE program is associated with intrinsic motivation and psycho-emotional development in primary students, and supports the feasibility of incorporating this type of innovative model into ordinary PE practice as a basis for more rigorous future research. These findings are in line with the idea that, using innovative models, PE may contribute to socio-emotional goals and life skills that transcend the school environment (Baena-Extremera et al., 2025).

## Figures and Tables

**Table 1:** Sample distribution by school.

**Gender**	**Alfredo Muiños Remolinos**	**Ángel Carrillo**	**Camacho Melendo**	**Campos Castellanos**	**Villas del Tajo**	**Maestro Juan Apresa**
Boys	78.6%	50.0%	53.2%	56.0%	39.4%	40.8%
Girls	21.4%	50.0%	46.8%	44.0%	60.6%	59.2%
Total	14	42	79	25	33	71
	**Ntr. Señora de la Asunción de Santacara**	**Princesa Sofía**	**Rodríguez Vega**	**Vicente Aleixandre**	**Virgen del Castillo**	**Santo** **Domingo de Guzmán FESD**
Boys	60.0%	54.5%	52.6%	51.4%	53.3%	46.8%
Girls	40.0%	45.5%	47.4%	48.6%	46.7%	53.2%
Total	10	66	38	37	30	47

**Table 2:** Means, standard deviations, skewness and kurtosis of variables in pre-test and post-test for boys.

	M	SD	γ1	γ2
Intrinsic motivation pre-	5.84	0.79	−0.45	−0.12
Intrinsic motivation post-	6.34	0.82	−1.70	3.35
Enjoyment pre-	3.96	0.58	−0.66	1.44
Enjoyment post-	4.35	0.55	−0.82	0.49
Self-confidence pre-	3.19	0.61	−0.63	−0.227
Self-confidence post-	3.55	0.47	−1.69	3.329
Resilience pre-	2.87	0.50	−0.73	1.159
Resilience post-	3.13	0.58	−0.80	0.094

Note: γ1 = skewness; γ2 = kurtosis.

**Table 3:** Means, standard deviations, skewness and kurtosis of variables in pre-test and post-test for girls.

	M	SD	γ1	γ2
Intrinsic motivation pre-	5.80	0.81	−0.11	−0.83
Intrinsic motivation post-	6.21	0.97	−1.50	1.88
Enjoyment pre-	3.92	0.53	−0.52	1.81
Enjoyment post-	4.30	0.58	−0.94	0.96
Self-confidence pre-	3.12	0.55	−0.40	−0.61
Self-confidence post-	3.47	0.52	−1.62	2.97
Resilience pre-	2.85	0.45	−0.76	0.94
Resilience post-	3.04	0.65	−0.97	0.34

Note: γ1 = skewness; γ2 = kurtosis.

**Table 4:** Correlation analysis for boys. Upper part: pre-test; lower part: post-test.

	Intrinsic.M	Enjoyment	Self-Confidence	Resilience
Intrinsic.M	-	0.520 **	0.301 **	0.306 **
Enjoyment	0.667 **	-	0.352 **	0.390 **
Self-Confidence	0.423 **	0.426 **	-	0.450 **
Resilience	0.436 **	0.344 **	0.503 **	-

Note: ** *p* < 0.01.

**Table 5:** Correlation analysis for girls. Upper part: pre-test; lower part: post-test.

	Intrinsic.M	Enjoyment	Self-Confidence	Resilience
Intrinsic.M	-	0.414 **	0.220 **	0.218 **
Enjoyment	0.736 **	-	0.160 **	0.165 **
Self-Confidence	0.538 **	0.463 **	-	0.467 **
Resilience	0.542 **	0.470 **	0.661 **	-

Note: ** *p* < 0.01.

**Table 6:** Block regression analysis in boys for pre- and post-treatment.

PRE BOYS						POST BOYS					
Intrinsic Motivation						Intrinsic Motivation					
Variables	F	β	R^2^	t	*p* (95% CI)	Variables	F	β	R^2^	t	*p* (95% CI)
Enjoyment	91.44	0.710	0.270	9.56	0.000 (0.564; 0.857)	Enjoyment	198.39	0.997	0.445	14.09	0.000 (0.858; 1.136)
Enjoyment + Self-confidence	49.29	0.646 0.175	0.286	8.21 2.34	0.000 (0.491; 0.801) 0.020 (0.028; 0.322)	Enjoyment + Self-confidence	107.50	0.891 0.283	0.462	11.51 3.11	0.000 (0.739; 1.04) 0.002 (0.103; 0.462)
Enjoyment + Self-confidence + Resilience	33.55	0.615 0.136 0.132	0.283	7.53 1.70 1.31	0.000 (0.454; 0.776) 0.090 (−0.021; 0.294) 0.184 (−0.063; 0.327)	Enjoyment + Self-confidence + Resilience	74.35	0.838 0.202 0.173	0.477	10.40 2.072 2.183	0.000 (0.679; 0.997) 0.039 (0.010; 0.394) 0.030 (0.017; 0.330)

**Table 7:** Block regression analysis in girls for pre- and post-treatment.

PRE GIRLS						POST GIRLS					
Intrinsic Motivation						Intrinsic Motivation					
Variables	F	β	R^2^	t	*p* (95% CI)	Variables	F	β	R^2^	t	*p* (95% CI)
Enjoyment	49.81	0.635	0.171	7.05	0.000 (0.458; 0.812)	Enjoyment	285.14	0.736	0.540	16.87	0.000 (1.08; 1.37)
Enjoyment + Self-confidence	29.21	0.596 0.233	0.200	6.66 2.70	0.000 (0.419; 0.773) 0.007 (0.063; 0.403)	Enjoyment + Self-confidence	173.72	0.620 0.251	0.588	13.32 5.40	0.000 (0.885; 1.19) 0.000 (0.295; 0.634)
Enjoyment + Self-confidence + Resilience	20.40	0.581 0.165 0.185	0.204	6.44 1.71 1.56	0.000 (0.404; 0.759) 0.088 (−0.025; 0.355) 0.120 (−0.048; 0.418)	Enjoyment + Self-confidence + Resilience	122.13	0.586 0.160 0.161	0.600	12.39 2.87 2.88	0.000 (0.826; 1.14) 0.004 (0.093; 0.499) 0.004 (0.076; 0.405)

**Table 8:** Inter-subject effects.

Effect	F	df	*p*	η^2^p	Power
Time	80.76	1, 490	<0.001	0.141	1.00
Time × Gender	0.69	1, 490	0.406	0.001	0.132
Gender	2.37	1, 490	0.124	0.005	0.336

**Table 9:** Marginal means.

Gender	Pre-Test M (95% CI)	Post-Test M (95% CI)
Boys	5.84 [5.74–5.94]	6.34 [6.23–6.45]
Girls	5.79 [5.69–5.89]	6.21 [6.09–6.32]

## Data Availability

The data presented in this study are available on request from the corresponding author. The data are not publicly available due to privacy restrictions.
